# Plasma Glycated and Oxidized Amino Acid-Based Screening Test for Clinical Early-Stage Osteoarthritis

**DOI:** 10.3390/antiox14101146

**Published:** 2025-09-23

**Authors:** Aisha Nasser J. M. Al-Saei, Usman Ahmed, Edward J. Dickenson, Kashif Rajpoot, Mingzhan Xue, Essam M. Abdelalim, Abdelilah Arredouani, Omar M. E. Albagha, Damian R. Griffin, Paul J. Thornalley, Naila Rabbani

**Affiliations:** 1 Department of Basic Medical Science, College of Medicine, QU Health, Qatar University, Doha P.O. Box 2713, Qatar; 2 Worcestershire Acute Hospitals NHS Trust, Charles Hastings Way, Worcester WR5 1DD, UK; 3 University Hospitals of Coventry & Warwickshire NHS Trust, Clifford Bridge Road, Coventry CV2 2DX, UKdamian.griffin@warwick.ac.uk (D.R.G.); 4 School of Computer Science, University of Birmingham Dubai, Dubai International Academic City, Dubai P.O. Box 341799, United Arab Emirates; 5 Qatar Biomedical Research Institute, Hamad Bin Khalifa University, Qatar Foundation, Doha P.O. Box 34110, Qatar; mxue@hbku.edu.qa (M.X.);; 6 Clinical Sciences Research Laboratories, Warwick Medical School, University of Warwick, University Hospitals Coventry and Warwickshire, Clifford Bridge Road, Coventry CV2 2DX, UK

**Keywords:** osteoarthritis, machine learning, glycation, oxidative stress

## Abstract

The diagnosis of early-stage osteoarthritis (eOA) is important in disease management and outcomes. Herein we report the clinical validation of a blood test for the diagnosis of eOA in a large patient cohort using trace-level glycated and oxidized amino acid analytes. Subjects were recruited and enrolled in two study groups: subjects with eOA of the hip (n = 110) and asymptomatic controls (n = 120). Their plasma was analyzed for glycated and oxidized amino acids by quantitative liquid chromatography–tandem mass spectrometry. Algorithms were developed using plasma hydroxyproline and 12 glycated and oxidized amino acid analyte features to classify the subjects with eOA and asymptomatic controls. The accuracy was defined as the percentage of the subjects correctly classified in the test set validation. The minimum number of analyte features required for the optimum accuracy was five glycated amino acid analytes: N_ω_-carboxymethyl-arginine, hydroimidazolones derived from glyoxal, methylglyoxal and 3-deoxyglucosone, and glucosepane. The classification performance metrics included an accuracy of 95%, sensitivity of 96%, specificity of 94%, area under the curve of the receiver operating characteristic curve of 99%, and positive and negative predictive values of 94% and 97%. We concluded that an assay of five trace-level glycated amino acids present in plasma can provide a simple blood test for the screening of eOA. This is predicted to improve the case identification for expert referral 9-fold.

## 1. Introduction

Osteoarthritis (OA) is a common musculoskeletal disease and the leading cause of pain and dysfunction, particularly in the older and overweight population [1,2]. It is a progressive joint disease, often with a long pre-symptomatic phase. Mechanistically, it is characterized by joint inflammation, proteolysis, and a reparative bone response—the latter is eventually overwhelmed as insidious chronic pain and the impairment of joint mobility develop. The key skeletal sites of impact are the joints of the knee and hip, which, with progression to severe symptoms, leads to the need for joint replacement surgery. Patients’ quality of life is greatly impaired by the restriction of their joint movement and chronic pain associated with OA development and its progression to severe symptoms [3,4].

The detection of early-stage osteoarthritis (eOA) prior to the development of severe symptoms would offer opportunities for lifestyle interventions and therapeutic development to prevent disease progression [5]. Effective early-stage treatment would also decrease the burden on healthcare services for the care of patients with OA [6]. There is no generally applicable and approved biochemical test for the diagnosis of eOA. Conventional radiographic image analysis detects joint abnormalities only in advanced and severe OA when irreversible damage has occurred. Biomarkers for the detection of eOA and other early-stage arthritic diseases are in development, including pre-symptomatic cartilage texture maps of joints created by magnetic resonance imaging [7] and multiplex immunoassays, as have recently been reviewed [8]. A simple clinical chemistry blood test with the capability for implementation on a generally available analytical platform would provide widespread access to eOA screening for effective and timely diagnosis.

We previously reported the discovery phase of a blood test for early-stage arthritis of different types based on the quantitation of trace-level glycated and oxidized amino acids [9,10]. Glycated and oxidized amino acids are released by the proteolysis of cartilage and other joint proteins in early-stage arthritic disease. They readily equilibrate from the joint to plasma and are not re-incorporated into proteins, so the plasma levels are very sensitive to changes in the rate of release from the joint. Plasma glycated and oxidized amino acids have been conveniently and robustly measured through stable isotope dilution analysis employing liquid chromatography–tandem mass spectrometry (LC-MS/MS)—an established clinical chemistry analysis platform [11]. The diagnostic utility of this method has been enhanced through the development of classifier algorithms employing glycated and oxidized amino acids as features using machine learning [9].

Herein we report a clinical validation study of this prospective blood test for the diagnosis of early-stage arthritis in a large, independent patient cohort, with applications to eOA.

## 2. Methods

### 2.1. Subjects with Early-Stage Osteoarthritis, Asymptomatic Control Subjects, Blood Sampling, and Analysis

The subjects for the study were recruited and enrolled at Meridan Hospital, Coventry, UK, and Qatar Biobank, Doha, Qatar. One hundred and ten subjects were enrolled and recruited at Meridan Hospital and one hundred and twenty subjects were recruited from Qatar Biobank. The subject characteristics are given in Table 1.

The criteria for subjects with eOA were new-onset hip pain, normal radiographs of the symptomatic hip, and an arthroscopic examination showing macroscopic findings classified as grade I or II on the Outerbridge scale [13]. The subjects were undergoing an exploratory arthroscopic examination after expert referral as part of a clinical investigation to identify the cause of the new-onset hip pain. For asymptomatic control subjects, the inclusion criteria were no history of joint symptoms, arthritic disease, or other morbidities, and the exclusion criteria were a history of injury or pain in either hip, taking medication—except oral contraceptives and vitamins—and any abnormality identified upon a physical examination of the hip.

Peripheral venous blood samples were collected from the subjects after overnight fasting with an ethylenediaminetetra-acetic acid (EDTA) anti-coagulant. The concentrations of glycated and oxidized amino acids in plasma and hydroxyproline (hyp) were determined as previously described [9]. The samples were collected from the patients and asymptomatic control subjects with their informed consent.

The sample collection and use were approved by local medical ethics committees and conducted in accordance with the Declaration of Helsinki. Ethical approval for this work was sought and obtained from local medical ethics committees, the East Midlands—Derby Research Ethics Committee, UK (ref no. 16/EM/0095), Qatar Biomedical Research Institute Institutional Review Board (ref no. QBRI-IRB 2019-013), and Qatar Biobank Institutional Review Board (ref no. E-2020-QF-QBB-RES-ACC-0183-0108), Doha, Qatar. Informed consent was obtained from all the participants before their enrollment.

### 2.2. Machine Learning Analysis

We sought to develop classifier algorithms to distinguish between the subjects with eOA and asymptomatic controls [9]. Diagnostic algorithms were developed using a 5-fold cross-validation protocol: the algorithms were initially trained on 80% of the subject data—the training set—before being used to predict the eOA or asymptomatic control class for each subject for the remaining 20% of the subject data—the test set. The algorithms were developed using the Support Vector Machine method and a panel of 13 plasma biomarkers: hyp and 12 glycated and oxidized amino acids (Table 2).

We used the area under the curve of the receiver operating characteristic plot (AUROC) statistic as the measure of the classification performance [14], identifying the minimum set of features providing the maximum AUROC [9,10]. We determined the 95% CI of the classification performance parameters via bootstrap analysis using the R package “pROC” [15].

### 2.3. Statistical Analyses

Normally distributed variables were summarized as the mean ± the SD, and two groups were compared using Student’s *t*-test. Non-normally distributed variables were summarized as the median (lower–upper quartiles), and two groups were compared using the Mann–Whitney U test. A Bonferroni correction of 13 was applied for the testing of the amino acid analytes, assuming a null hypothesis that the levels of any of the 13 analytes measured may have changed significantly in patients with eOA compared to the asymptomatic controls. The data were significantly different when *p* < 0.05/13 or <0.0038. This report adheres to the guidelines for the transparent reporting of a multivariable prediction model for an individual prognosis or diagnosis (TRIPOD) statement [16].

## 3. Results

### Classification of Subjects with Early-Stage Osteoarthritis and Asymptomatic Controls Using Algorithms Developed Using Plasma Glycated and Oxidized Amino Acids and Hydroxyproline as Features

The concentrations of 12 glycated and oxidized amino acids and hyp in plasma were determined in the subjects with and without eOA. All the analytes were increased significantly in the subjects with eOA except for dityrosine (DT) and glyoxal-derived hydroimidazolone (G-H1) and N_ω_-carboxymethyl-arginine (CMA), which were decreased. The results remained significant after Bonferroni correction (Table 2).

**Figure 1:**
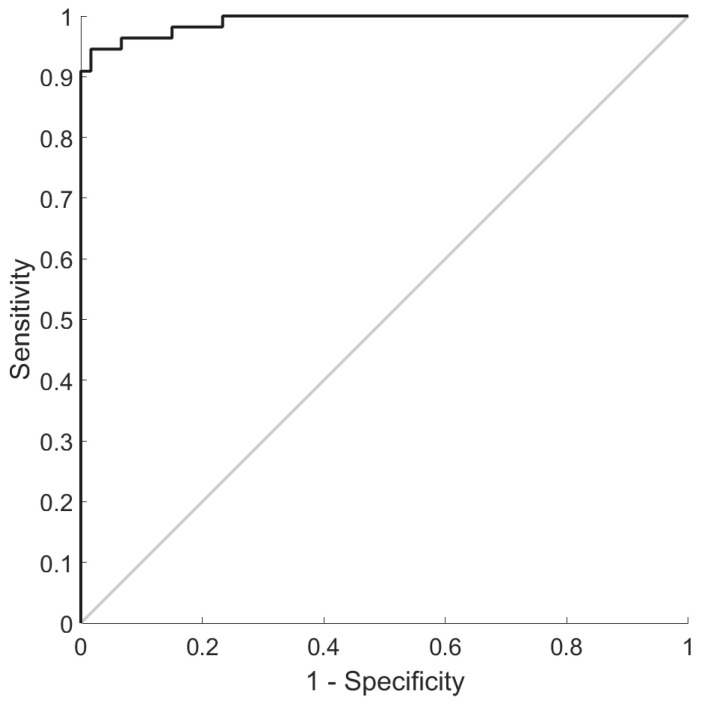
Receiver operating characteristic curves for the training set with an AUROC of 0.99 (0.99–1.00).

In this validation study, the minimum features in the diagnostic algorithms providing the optimum classification of subjects with and without eOA were the plasma concentrations of five glycated amino acids: CMA, G-H1, hydroimidazolones derived from methylglyoxal and 3-deoxyglucosone (MG-H1 and 3DG-H, respectively), and glucosepane (GSP). The classification accuracy was 95%, the sensitivity was 96%, the specificity was 94%, and the AUROC was 99% (Figure 1). The positive and negative predictive values, the PPVs and NPVs, were 94% and 97%, and the positive and negative likelihood ratios, the LR+s and LR−s, were 21.4 and 0.04, respectively, indicating that the test provides strong, often convincing evidence of the presence and absence of eOA (Table 3).

## 4. Discussion

It has long been known that protein glycation and oxidation adducts accumulate in the cartilage of the knee and hip joints with age [17,18,19] and the proteolysis of cartilage occurs in the early-stage development of OA [20]. The blood test herein exploits this to provide diagnostic biomarkers for eOA. In previous studies we found that similar algorithms including the anti-cyclic citrullinated peptide (CCP) antibody status as a feature also provided the high-accuracy classification of early-stage rheumatoid arthritis (eRA). So, similar algorithm-based classification of eRA may be possible using glycated and oxidized amino acids, along with the anti-CCP antibody status, as features.

In this validation study, the median concentrations of the plasma free G-H1 and CMA adducts were lower in the subjects with eOA than in the asymptomatic controls. This was not found in the discovery phase study in [9]. G-H1 and CMA adducts are formed from the proteolysis of proteins modified by glyoxal, which mainly originates from lipid peroxidation [11]. This may indicate that the level of lipid peroxidation is a potential confounder in this diagnostic application. The decreased levels of lipid peroxidation-linked protein adducts in the subjects with eOA may reflect the recent uptake of dietary guidance for those at risk of osteoarthritis to eat a diet rich in fruits, vegetables, fish, whole grains, and legumes containing antioxidants [21] that decrease lipid peroxidation, although eOA had still developed, likely driven by other risk factors.

The test has typical characteristics preferred for a clinical blood test methodology: a small sample size (50 µL of plasma), a relatively short analysis time (ca. 30 min), good internal validity, external validity, test–retest reliability, and inter-rater reliability. It is based on stable isotopic dilution analysis employing LC-MS/MS, an analytical technique which is the gold-standard reference technique for such small-molecule quantitation with high analytical sensitivity and specificity, robust calibration, and good reproducibility [11]. LC-MS/MS is often preferred for the harmonization of analytic measurements between laboratories [22] and is regarded by the USA Food and Drug Administration as an appropriate analytical technology for use in clinical in vitro diagnostic tests [23], such as a test for the diagnosis of eOA.

This blood test provides a simple clinical chemistry methodology for the identification of subjects with and without eOA with high classification accuracy based on putative biochemical markers of proteolysis in the affected joint. We propose that this blood test may be optimally used as a clinical screening test, identifying subjects for referral to orthopedic consultants for expert assessment and care. For the prediction of its performance in clinical use, we assume that the population prevalence of eOA in subjects ≥ 60 years old is ca. 20% [24]. The implementation of a simple OA pre-screening questionnaire with a reported sensitivity of 87% and specificity of 60% for eOA of the hip [25] increases the prevalence of eOA cases in patients with positive pre-screening outcomes to ca. 45%. With the application of this blood test to patients with positive outcomes from pre-screening with the classification sensitivity and specificity found herein, the population screening PPV increases to 93% and the NPV to 95%. This indicates that the false discovery rate and false omission rate of eOA cases after the blood test would be very low, 7% and 5%, respectively. Indeed, these are decreased ca. 9-fold compared with the rates with the pre-screening questionnaire alone (65% and 46%). The implementation of the blood test would, therefore, greatly improve the effectiveness of eOA case detection for expert referral and thereby the effective use of orthopedic consultants’ time and would likely reduce the cost to national health services supporting these patients.

The increased formation and accumulation of advanced glycation endproducts (AGEs) in arthritis and a link to the decreased metabolism of glyoxal and MG with decreased glyoxalase 1 (Glo1) [26]—which is involved in the main pathway for the metabolism of these reactive dicarbonyl metabolites—have long been suspected. Increased skin and urinary pentosidine levels in patients with mild radiographic joint damage and low versus high cartilage breakdown were found in OA, suggesting that AGEs may contribute to susceptibility to OA and/or OA progression [27]. In a comprehensive study of protein glycation, oxidation, and nitration adducts, we previously reported the presence of glycated, oxidized, and nitrated proteins and amino acids in the synovial fluid and plasma of patients with eOA, with the modified amino acids increasing further in severe and advanced OA [9]. In an animal model of OA, the plasma levels of GSP free adduct correlated positively with the OA severity and joint cartilage stiffness, and the plasma DT free adduct concentration correlated positively with the OA severity. Interleukin-1β increased the release of glycated and nitrated amino acids from chondrocytes in vitro [10]. Glo1 expression and activity was impaired by inflammation mediated by IL-1β in chondrocytes [28]. The finding that CMA, G-H1, MG-H1, 3DG-H, and GSP were the minimum features required in a diagnostic algorithm for eOA in this study is consistent with increased reactive dicarbonyl metabolites, dicarbonyl stress [26], and glycation crosslinking changes being key mechanistic features of early-stage joint derangement.

## 5. Conclusions

From this validation study, we conclude that the measurement of five trace-level glycated amino acids in plasma by LC-MS/MS and their combined use as biomarker features in diagnostic algorithms provides a simple blood test with high accuracy, sensitivity, and specificity for the screening of eOA.

## Figures and Tables

**Table 1:** Clinical characteristics of the participants.

Study Group	N	Age (yr)	Gender (M/F)	BMI (kg/m^2^)	iHOT-33
Asymptomatic controls	120	33 ± 9	60/60	28 ± 5	
Subjects with eOA	110	39 ± 12	54/56	25 ± 6	39 ± 17

Data are mean ± SD. Abbreviations: BMI, body mass index; iHOT-33, International Hip Outcome Tool 33—score assessing severity of symptoms of OA of hip in relatively young and active patients with hip disorders [12].

**Table 2:** Concentrations of hydroxyproline and glycated and oxidized amino acids in plasma of subjects with early-stage osteoarthritis and asymptomatic controls.

	[Analytes]_Plasma_ (nM)		
Analyte	Asymptomatic Controls (n = 120)	eOA (n = 110)	*p*-Value
Hydroxyproline (Hyp)	340 (70–1870)	1570 (45–6440)	<0.001 ***
N_ε_-Fructosyl-lysine (FL)	543 (170–2770)	262 (79.1–12,259)	<0.001 ***
N_ε_-Carboxymethyl-lysine (CML)	77 (29–209)	50.4 (32.5–87.5)	<0.001 ***
N_ε_(1-Carboxyethyl)lysine (CEL)	145 (27–543)	58.5 (20.0–146.0)	<0.001 ***
Glyoxal-derived hydroimidazolone (G-H1)	3.53 (1.27–7.27)	2.66 (0.366–5.86)	<0.001 ***
Methylglyoxal-derived hydroimidazolone (MG-H1)	446 (104–2343)	614 (142–3502)	0.014 *
3-Deoxyglucosone-derived hydroimidazolone (3DG-H)	70 (37–262)	143 (130–501)	<0.001 ***
N_ω_-Carboxymethyl-arginine (CMA)	24.6 (9.3–86.9)	19.4 (5.22–45.8)	<0.001 ***
Glucosepane (GSP)	17.3 (6.7–86.7)	28.4 (6.81–64.9)	<0.001 ***
Methionine sulfoxide (MetSO)	95 (30–1118)	199 (44.3–944)	<0.001 ***
Dityrosine (DT)	0.81 (0.44–1.28)	0.835 (0.276–1.72)	
N-Formyl-kynurenine (NFK)	6.64 (0.104–270)	10.0 (1.03–62.0)	<0.01 **
3-Nitrotyrosine (3-NT)	1.10 (0.13–1.63)	1.68 (0.302–4.98)	<0.001 ***

The data are the medians (lower–upper quartiles). Significance (Mann–Whitney U): *, **, and *** represent *p* < 0.05, *p* < 0.01, and *p* < 0.001 after a Bonferroni correction of 13 was applied.

**Table 3:** Features and performance of the diagnostic algorithm for classification of early-stage osteoarthritis patients versus asymptomatic controls.

Features	CMA, G-H1, MG-H1, 3DG-H, and GSP Free Adducts
nCorrect	219/230
Sensitivity (%)	96 (95–98)
Specificity (%)	94 (91–98)
AUROC (%)	99 (99–100)
Positive predictive value (%)	94
Negative predictive value (%)	97
Positive likelihood ratio (LR+)	21.4
Negative likelihood ratio (LR−)	0.04

Data are means (95% CI where given). Data were analyzed using R version 3.1.3.

## Data Availability

The data from this study are available from the corresponding author upon reasonable request.
